# Covalent Bi-Modular Parallel and Antiparallel G-Quadruplex DNA Nanocostructs Reduce Viability of Patient Glioma Primary Cell Cultures

**DOI:** 10.3390/ijms22073372

**Published:** 2021-03-25

**Authors:** Valeria Legatova, Nadezhda Samoylenkova, Alexander Arutyunyan, Vadim Tashlitsky, Elena Zavyalova, Dmitry Usachev, Galina Pavlova, Alexey Kopylov

**Affiliations:** 1 Chemistry Department, Lomonosov Moscow State University, 119991 Moscow, Russia; legatovav.chem.msu@gmail.com (V.L.); zlenka2006@gmail.com (E.Z.); 2 Burdenko Neurosurgical Institute, 125047 Moscow, Russia; samoylenkova.n@gmail.com (N.S.); dousachev@nsi.ru (D.U.); 3 Belozersky Research Institute of Physical Chemical Biology, Lomonosov Moscow State University, 119991 Moscow, Russia; ararut@belozersky.msu.ru (A.A.); tashlitsky@belozersky.msu.ru (V.T.); 4 Institute of Higher Nervous Activity and Neurophysiology, Russian Academy of Sciences, 117485 Moscow, Russia; 5 Department of Medical Genetics, Sechenov First Moscow State Medical University, 119991 Moscow, Russia

**Keywords:** G-quadruplexes, covalent dimer construct, anti-proliferative activity, primary cell culture of human glioma

## Abstract

G-quadruplex oligonucleotides (GQs) exhibit specific anti-proliferative activity in human cancer cell lines, and they can selectively inhibit the viability/proliferation of cancer cell lines vs. non-cancer ones. This ability could be translated into a cancer treatment, in particular for glioblastoma multiform (GBM), which currently has a poor prognosis and low-efficiency therapeutic treatments. A novel bi-modular GQ, bi-(AID-1-T), a twin of the previously described three-quartet AID-1-T, was designed and studied in terms of both its structure and function. A covalent conjugation of two AID-1-Ts via three thymidine link, TTT, did not interfere with its initial GQ structure. A comparison of bi-(AID-1-T) with its mono-modular AID-1-T, mono-modular two-quartet HD1, and bi-modular bi-HD1, as well as conventional two-quartet AS1411, was made. Among the five GQs studied, bi-(AID-1-T) had the highest anti-proliferative activity for the neural cancer cell line U87, while not affecting the control cell line, human embryonic fibroblasts. GQs, for the first time, were tested on several primary glioma cultures from patient surgical samples. It turned out that the sensitivity of the patient primary glioma cultures toward GQs varied, with an apparent IC_50_ of less than 1 μM for bi-(AID-1-T) toward the most sensitive G11 cell culture (glioma, Grade III).

## 1. Introduction

G-quadruplex DNAs (GQs) have been well-known anti-proliferative agents since the beginning of the millennium [1]. A straightforward hypothesis, which could explain this effect, assumes that some regulatory regions of genomic DNA have GQ structures that are involved in the regulation of cell proliferation [2]. Therefore, after cellular uptake, GQ could bind with regulatory proteins (GQ-binding proteins), competing with regulatory GQ DNA regions, and therefore providing pleiotropic effects; anti-proliferation included. Knowing a target DNA regulatory region, a rational approach for a functional GQ design has been developed: GQ DNA decoys. For example, oligonucleotides mimicking a particular GQ motif in the KRAS promoter were found to compete with DNA–protein complexes between non-homologous end joining (NHE) and a nuclear extract from pancreatic cancer cells [3].

Moreover, a current approach is the screening of random GQs [1], no matter which way the GQ was originated. Both GQ DNA structures and the type of sensitive cells vary, therefore requiring the application of empirical approaches for screening the activity of different GQs toward different types of cells to establish an effective “corresponding pair” to study further in more detail. Additional studies will be required to answer the question of whether and how the GQ structures of oligonucleotides contribute to their cellular uptake and anti-proliferative activity [4]. Here are three the most successful examples:

The first example of anti-proliferative GQ is AS1411, developed by Bates et al. since 2009 [5] as the first-in-class anti-cancer GQ. A tumor-targeting aptamer AS1411 is a 26-mer DNA oligonucleotide that has a polymorphic GQ structure, and binds to nucleolin, which is preferentially expressed on the surface of cancer cells [6]. AS1411 provides cancer cell cytotoxicity [7] by causing methuosis, a novel type of non-apoptotic cell death, via nucleolin stimulated Rac1 activation, and massive vacuolization in the cytoplasm [8]. Therefore, AS1411 potentially serves as an anti-cancer therapeutic agent. AS1411 has been evaluated in phase II clinical trials for acute myeloid leukemia and renal cell carcinoma [9]. It exhibited a good safety profile, but the trial was terminated because of poor pharmacokinetics, requiring the enhancement of stability in circulation for a further translation [6]. In addition, it could serve as a tumor-targeting agent. For example, recently a novel targeted drug delivery system (TDDS) with practical application potential for colon cancer treatment has been developed. The TDDS was built by loading docetaxel in albumin nanoparticles that were functionalized with AS1411 [10].

**Figure 1:**
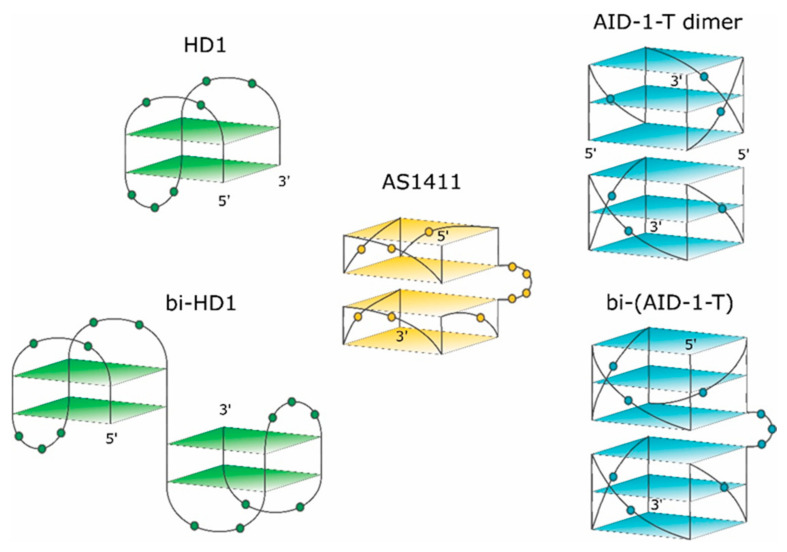
Schematic pictures of putative structures of the GQs under study. The 3-D structure of HD1 is known. 5′- and 3′-ends are indicated. Nucleotides are shown in the loops only.

Conformational polymorphism could be a negative factor for successful translation [11]. To reduce polymorphism, a new AS1411 derivative, AT11, has been synthesized with a unique GQ conformation. It turned out that it has two covalently conjugated propeller-type parallel-stranded two-tetrad modules [4] (Figure 1). AT11 has an anti-proliferative activity, like AS1411, for human lung cancer cell line, and does not affect the normal cell lines.

Recently, some more derivatives of AS1411 with anti-proliferative activity toward MCF-7 cells have been developed [12]. GQs with three-tetrad structures were 10 times more active than the initial AS1411 with two-tetrad GQ.

The second example of an anti-proliferative GQ is HD1, which came from a different area. Originally, 15-mer DNA GQ HD1 (Figure 1) was discovered in 1991 as a thrombin binding aptamer via selection by conventional SELEX [13]. As HD1 has a typical GQ structure, it exhibits anti-proliferative activity like some other GQs [1,14,15].

The third example of an anti-proliferative GQ is a T-series GQ DNA, which has simple repeating sequences like (G3T)_4_ for T30923 [16] (Figure 1) for (G3T)_4_ -T), and (G3C)_4_ for T40214 [17].

Recently, some rational designs for making anti-proliferative GQ have been developed by us. Attempting to enhance the anti-proliferative activity, two single GQ DNA modules were covalently conjugated to make a bi-modular, “twin” molecule (see also [18]). Certainly, a simple covalent joining of two single GQ modules does not necessarily yield a perfect “twin” molecule with a double anti-proliferative activity, because extensions at both the 3′- and 5′-ends could affect the GQ stability and functioning [19]. For instance, HD1 could be covalently conjugated via a single T nucleotide; yielding bi-HD1 (Figure 1), which retains anti-proliferative activity [14].

This paper pursued this approach to make twin molecules and to explore their specific properties. The paper describes some structural and functional properties of a constructed, bi-(AID-1-T), bi-modular anti-proliferative GQ twin molecule, which was made by conjugating two modules of (G3T)_3_GGG via three T nucleotides (Figure 1).

## 2. Results

### 2.1. Topology of GQs and Bi-GQs

The topology of G-quadruplex oligonucleotides (GQs) was analyzed by characteristic circular dichroism (CD) spectroscopy. The CD spectrum of a parallel GQ had a huge positive band at 260 nm, a relatively shallow negative band at 240 nm, and another large positive band at 210 nm. The CD spectrum of an antiparallel quadruplex had a typical positive band at 295 nm, two other smaller ones at 240 and 210 nm, and a negative band at 260 nm [20,21,22,23].

AS1411, 26-mer GGTGGTGGTGGTTGTGGTGGTGGTGG, could form both monomolecular and bimolecular GQs. The 3-D structure of AS1411 was highly polymorphic in solution, with at least eight different GQs structures detected by chromatography and NMR [11,24]. The structure of AT11, 28-mer, with a close but mutated sequence (underlined) TGGTGGTGGTTGTTGTGGTGGTGGTGGT, turned out to have a single GQ conformation, and exhibited a similar anti-proliferative activity to AS1411. The solution structure of AT11, solved by NMR, revealed two GQs; each GQ is a propeller-type parallel-stranded two-tetrad module (Figure 1). The covalent conjugation supports stacking via the 3′-5′ interface [4].

HD1, 15-mer GGTTGGTGTGGTTGG, folds into antiparallel GQ with chair-like conformation (Figure 1, for rev. [13]).

The exact 3-D structure of AID-1-T, 15-mer GGGTGGGTGGGTGGG [25], is not yet known. AID-1, a close counterpart of AID-1-T (also coined as T30923), has an additional T at the 3′-end; its derivative, J19, with the single replacement G2I, GIGTGGGTGGGTGGGT, could form a noncovalent dimer: two identical propeller-type parallel-stranded three-tetrad GQ modules stacked via the 5′-5′ interface (Figure 1) [16].

**Figure 2:**
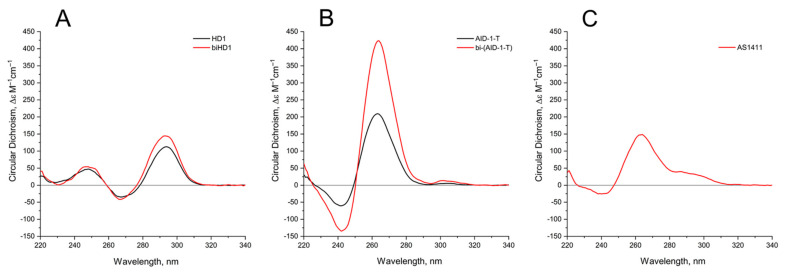
Circular dichroism (CD) spectra of the GQs under study in the buffer B (140 mM NaCl and 10 mM KCl) at 20 °C: (**A**) HD1 (black), bi-HD1 (red); (**B**) AID-1-T (black), bi-(AID-1-T) (red); (**C**) AS1411.

The 3-D structures of the covalent dimers discussed in the text, the twin molecules, are not known yet. The idea that, after covalent joining of two GQ modules, the twins have to keep the original structure of the initial modules is not obvious. On the contrary, it was shown that just the single stranded oligonucleotide extensions at both the 3′- and 5′-ends of GQ of HD1 could affect the GQ properties [19]. Indeed, for the simplest case, bi-HD1, it seems that the two GQ modules are not equal, being covalently conjugated via single T ([14], and refs therein). Indeed, the molar ellipticity of CD spectrum at 295 nm of bi-HD1 is not exactly twice as for HD1, but just 1.5 times (Figure 2A).

Continuing to explore the structural and functional properties of twin molecules, we made another twin, a novel bi-modular GQ, bi-(AID-1-T), a covalent dimer of AID-1-T module [25], connected with a TTT linker (Figure 1).

Comparing the spectra of mono-modular AID-1-T and covalent bi-modular GQ, bi-(AID-1-T), it is clear, that bi-GQ retains a topology of parallel GQ (Figure 2B). Moreover, the molar ellipticity of the covalent twin molecule is two times the ellipticity of the mono-modular one, despite the fact that AID-1-T does exist as a non-covalent dimer in the solution (see the next Section); therefore both molecules are dimers, either non-covalent AID-1-T or covalent bi-(AID-1-T). Though a striking difference is the following: Non-covalent dimerization of AID-1-T in solution happens via 5′-5′ stacking interactions [16], making the opposite orientation of two anti-parallel GQs, “face-to-face” (Figure 1). This is not the case for bi-(AID-1-T), where the only possibility is 5′–3′ stacking, due to the existence of the covalent link, making the same orientation of two anti-parallel GQs (Figure 1), and therefore yielding a twin with two-times higher ellipticity (Figure 2B).

As far as conformation of the reference molecule, AS1411, is concerned, it exhibits a spectrum for the mixture of conformers, as was shown before [11], with a prevailing parallel conformation (Figure 2C), which is in the vein of the suggested similarities with AT11 conformation [4].

GQ folding and stability strongly depend on the concentration and nature of the cations in a buffer [26,27,28]. Sodium cation Na^+^ is for a shielding the phosphate charges; potassium cation K^+^ is a very critical stabilizer of GQ structure, because it coordinates the octet of oxygen atoms of G-tetrads, and stabilizes GQ. Finally, barium cation Ba^2+^ is a very special coordination center, which arranges the same donors, the oxygen atoms of G, but much more strongly, and usually yielding a slightly different structure of GQ complex, unlike the one with K^+^ that could be clearly detected by CD spectroscopy [29].

**Figure 3:**
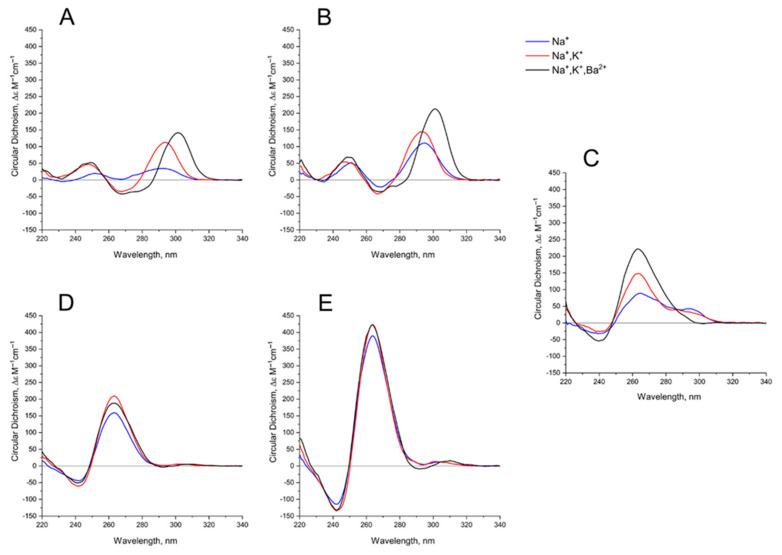
CD spectra GQs under study: in the buffer A (140 mM NaCl; (Na^+^), blue), in the buffer B (140 mM NaCl, 10 mM KCl; (Na^+^, K^+^), red), in the buffer C (140 mM NaCl, 10 mM KCl, 5 mM BaCl_2_; (Na^+^, K^+^, Ba^2+^), black) at 20 °C: (**A**) HD1, (**B**) bi-HD1, (**C**) AS1411, (**D**) AID-1-T, (**E**) bi-(AID-1-T).

The behavior of the CD spectra of mono-modular anti-parallel HD1 is as described previously [29], showing increasing folding efficiency when using K^+^ instead of Na^+^ coordination; Ba^2+^ has a bigger effect, yielding a shift of the CD maximum from 294 nm to 302 nm (Figure 3A). As far as mono-modular parallel GQ structures are concerned, AS1411, being a mixture of conformers, shows the same tendency, with an increasing proportion of folding into the parallel structure after K^+^ coordination, and almost perfect folding after Ba^2+^ coordination (Figure 3C). Not surprisingly, the folding of AID-1-T, as a perfect parallel GQ, is less affected by the nature of the cation (Figure 3D).

Bi-modular GQs are less sensitive to cation nature. Compared to HD1, bi-HD1 is folded sufficiently, even with Na^+^; not mentioning that in Ba^2+^ solution it seems that both GQ modules of bi-HD1 were perfectly folded (Figure 3B). The parallel bi-modular bi-(AID-1-T) folds perfectly, with no dependence on cation nature (Figure 3E).

### 2.2. Thermal Stability of GQs and Bi-GQs

**Figure 4:**
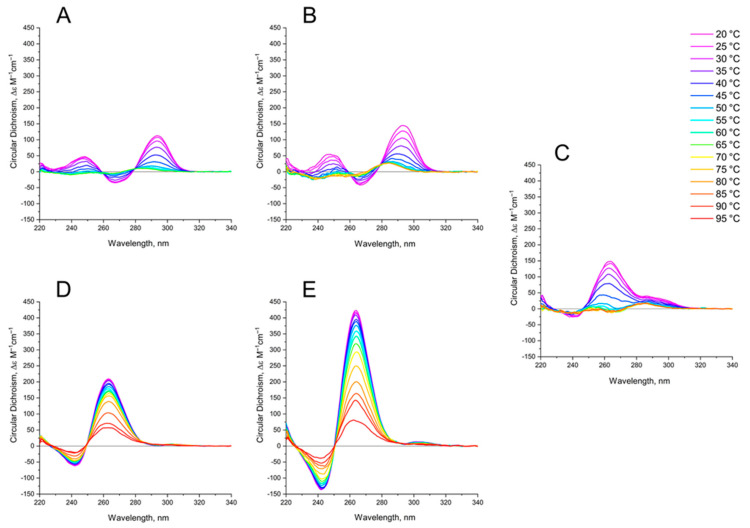
CD spectra of GQs under study in buffer B (140 mM NaCl and 10 mM KCl) at different Table 1. (**B**) bi-HD1; (**C**) AS1411 (**D**) AID-1-T, (**E**) bi-(AID-1-T). The color ladder on the right indicates temperatures.

The thermal stability of GQs was analyzed by characteristic CD spectroscopy (Figure 4).

**Figure 5:**
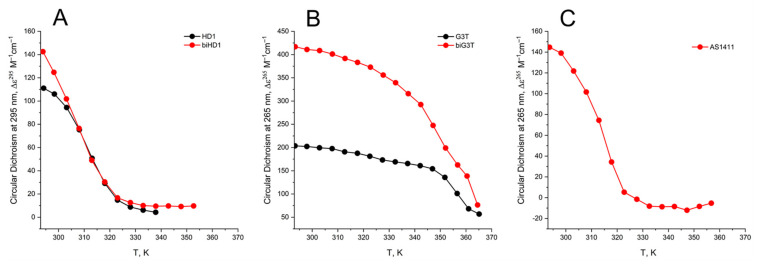
CD melting profile of GQs under study in buffer B (140 mM NaCl and 10 mM KCl): (**A**) HD1 (black), bi-HD1 (red); (**B**) AID-1-T (black), bi-(AID-1-T) (red); (**C**) AS1411 (red). Temperature is in kelvin (K): [°C] = [K] − 273.

The CD melting profile in the standard buffer B (140 mM and 10 mM KCl) of anti-parallel mono-modular HD1 was about the same as for bi-modular bi-HD1, as measured at 295 nm (Figure 5A). The apparent T*m* (aT*m*) were different, and about the same as those published earlier, 35 °C and 40 °C, respectively [14,30].

The aT*m* of parallel GQs were expectedly higher than the anti-parallel GQs. The melting behavior of mono-modular AID-1-T exhibited a biphasic profile, with two distinct transitions (Figure 5B): at about 50 °C, presumably a non-covalent dimer to monomer transition (see Sub-section “size-exclusion HPLC”); and at 83 °C, the unfolding of the parallel GQ module itself. The melting profile of the covalent dimer, bi-(AID-1-T), showed a less cooperative, gradual behavior (Figure 5B), while the CD-annealing profile showed no hysteresis.

Not surprisingly, the parallel AS1411, as a mixture of conformations, and considering its two-quartet nature, had a much lower aT*m*, about 40 °C, as measured at 265 nm (Figure 5C).

**Figure A1:**
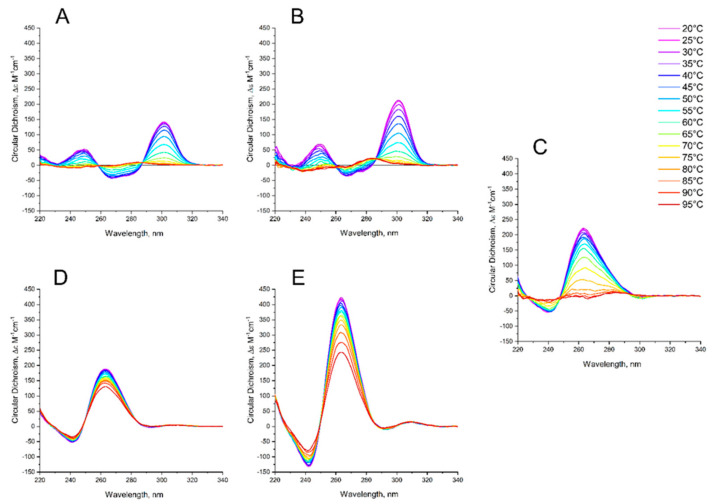
CD spectra of GQs under study in buffer C (140 mM NaCl, 10 mM KCl, 5 mM BaCl_2_) at different temperatures: (**A**) HD1, (**B**) bi-HD1; (**C**) AS1411, (**D**) AID-1-T, (**E**) bi-(AID-1-T). The color ladder on the right indicates temperatures.

Since the Ba^2+^ cation provides a very specific stabilizing effect, the melting of GQs in a buffer with Ba^2+^ was also studied (Figure A1). All tendencies of GQs melting Ba^2+^solutions, both for mono-module and bi-modular ones, were alike for the K^+^ solution, except the aT*m* were much higher.

### 2.3. Size-Exclusion HPLC of GQs and Bi-GQs

Size-exclusion HPLC (SE-HPLC) is a rather simple and powerful technique for the fast assessment of conformations and oligomeric forms of GQs, but it has not yet been widely applied [31] (for a review, see [32]). The separation matrix could be either polymethacrylate-based particles [32] or a silica-based one [24,33], which strongly affects the separation conditions.

Previously, we had developed conditions for silica-based SE-HPLC to perform separation with standard equipment [34,35], and applying a 10 µM concentration of GQ samples. Three different buffers were used (see CD Sub-section) to modulate the folding of GQs, and hence to estimate the conformational/oligomeric composition. It is worth mentioning that in our case, a good separation required a high salt concentration for the mobile phase; which could shift the equilibrium for dynamic conformers/oligomers if the pre-folding was made under different conditions.

**Figure 6:**
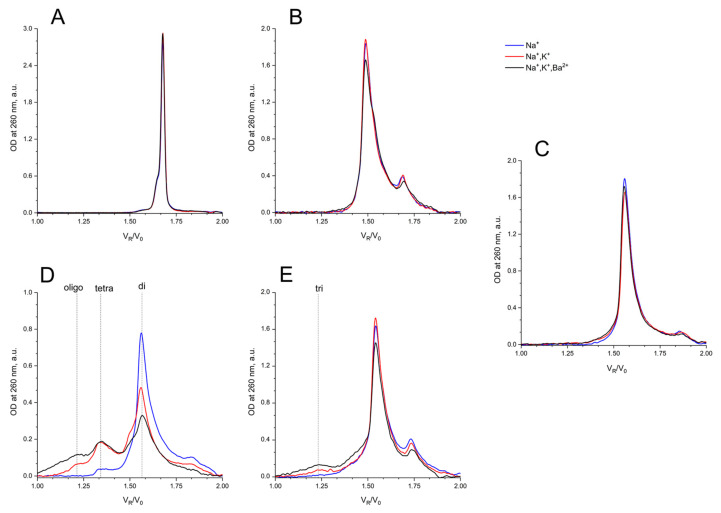
Size exclusion HPLC chromatography analysis of GQs under study: in buffer A (140 mM NaCl; (Na^+^), blue), in buffer B (140 mM NaCl, 10 mM KCl; (Na^+^, K^+^), red), in buffer C (140 mM NaCl, 10 mM KCl, 5 mM BaCl_2_; (Na^+^, K^+^, Ba^2+^), black) at 20 °C: (**A**) HD1, (**B**) bi-HD1, (**C**) AS1411, (**D**) AID-1-T, (**E**) bi-(AID-1-T).

Under all three conditions of pre-forming at high concentration, and with a subsequent transfer into separation conditions, AS1411 behaved as a dimer, and showing a slightly asymmetric peak (Figure 6C).

It is no surprise that the migration of bi-HD1 was much faster than mono-modular HD1. The bi-modular bi-(AID-1-T) exhibited approximately the same mobility as the non-covalent dimer of mono-modular AID-1-T; the dimerization ability of the later had also been observed before [16]. The introduction of Ba^2+^ stimulated further oligomerization of the non-covalent dimer.

### 2.4. Effect of GQs on the Viability of Cell Lines and Primary Glioma Cell Cultures from Patients

**Figure 7:**
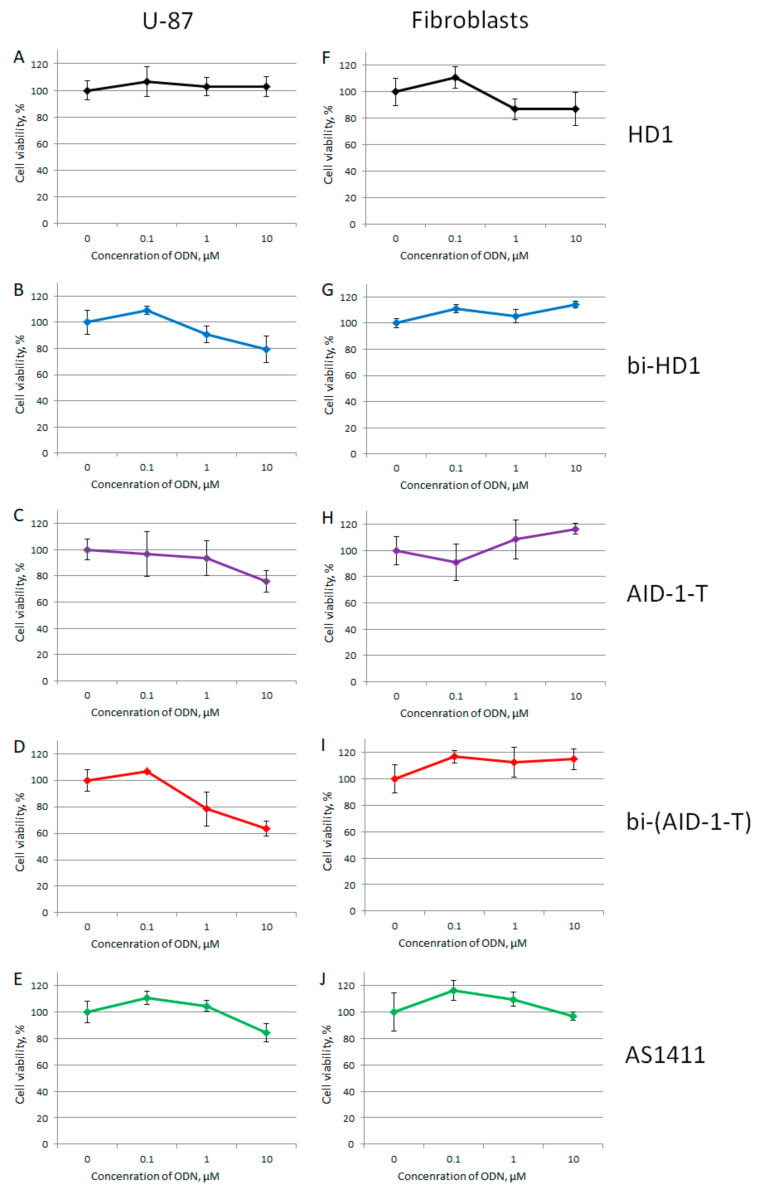
MTT assay data on the cell viability of two cell lines after GQ treatment with three different concentrations (0.1; 1.0, and 10 μM) for 72 h. Human cancer cell line U87 (**A**–**E**) and embryonic fibroblasts (**F**–**J**). HD1 (black), bi-HD1 (blue), AID-1-T (violet), bi-(AID-1-T) (red), AS1411 (green). Data are shown as (mean +/− S.D., standard deviation).

The focus of our study was the GBM primary cell lines from the patients, therefore the neural cancer cell line U87 was chosen as the reference, while fibroblasts from human embryo were the non-cancer controls. All GQs had no effect on the viability of fibroblasts from human embryos at all three concentrations tested: 0.1, 1, and 10 µM; whereas the effect on the viability of the reference U87 cell line strongly depended on the GQ structure (Figure 7).

Within the range from 1 to 10 µM of GQs, only three dimeric GQs had an effect, exhibiting a gradual decrease of cell viability: the two covalent ones, bi-HD1 and bi-(AID-1-T), and the non-covalent dimer, AID-1-T.

Despite the current wide application of cell lines for studying GBM, it has become more and more evident that these results cannot be translated directly to the behavior of primary cell cultures from patient samples (for example, see [36]). Therefore, in this research, six different primary glioma cell cultures from patient samples were studied. Some patient data are listed in Table 1.

**Figure 8:**
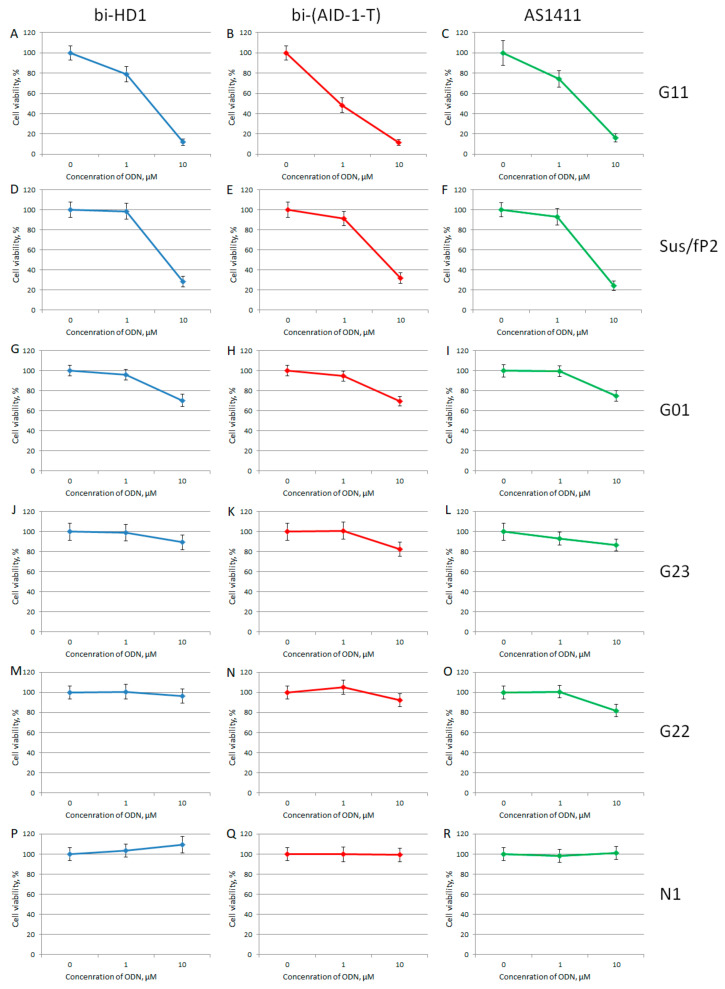
MTT assay data on the cell viability of six patient primary glioma cell cultures after bi-GQ treatment with three different concentrations (0.1; 1.0, and 10 μM) for 72 h. Patient primary glioma cell cultures: G11 (**A**–**C**), Sus/fP2 (**D**–**F**), G01 (**G**–**I**), G23 (**J**–**L**), G22 (**M**–**O**), N1 (**P**–**R**). bi-HD1 (blue), bi-(AID-1-T) (red), AS1411 (green). Data are shown as (mean +/− S.D.). Patient data are in the Table 1.

For studying the effect of GQs on the viability of cell cultures by MTT test, only twin molecules were selected, including a putative twin, the reference AS1411. Two out of six primary glioma cell cultures turned out to be sensitive toward bi-GQs treatment: G11 and Sus/fP2 (both derived from female samples) (Figure 8); and G11 was more sensitive than Sus/fP2. One other cell culture, G01, became sensitive to GQs only at a high concentration of 10 µM.

When comparing the activity of the two bi-GQs, bi-HD1 and bi-(AID-1-T), toward two sensitive cell cultures, G11 and Sus/fP2, bi-(AID-1-T) turned out to be the most active GQ, and could reduce viability twice, even at 1 µM GQ.

## 3. Discussion

GQs have a well-documented anti-proliferative activity [1]. Besides the numerous attempts at searching for more GQs with anti-proliferative activity, there are three established arms of research, which are based on the original mono-modular GQs, namely, AS1411, HD1, and the so called T-series.

AS1411 is a conventional GQ with anti-proliferative activity toward a variety of cell lines [6]. It is at the stage of translation, and we took it as the reference GQ. The disadvantage of AS1411 is its conformational heterogeneity, which was overcame using derivatives, like AT11 [5].

An idea for the further development of the other two arms of GQ anti-proliferative study is to conjugate known functional GQ modules, making a bi-modular twin molecule. We focused on the brain cancer, glioma. Most of the data for GQ anti-proliferative activity have been recorded using cancer cell lines, while this research was focused, for the first time, on patient glioma primary cell cultures.

Because of a lack in the literature of well-established conventional conditions for the treatment of cells with oligonucleotides, the critical point was choosing a threshold for screening. We restricted this value to 10 μM.

We have shown earlier [14] for the CNS cancer cell line, U87, that two-quartet GQ, bi-HD1, has an anti-proliferative activity; and that bi-modular constructed bi-HD1 is much more active than mono-modular HD1: GQ concentration below 10 μM GQ, for 72 h. Therefore, these GQs (Figure 1) were chosen as the reference to compare mono-modular and bi-modular GQs for anti-proliferative activity.

AID-1-T is a three-quartet GQ with an anti-proliferative activity toward a variety of cell lines [37]. Continuing the bi-modular construction, a bi-(AID-1-T) with TTT-linker in between two GQ modules was designed for the first time.

The three-dimensional structures of GQ modules are known for HD1 [38], and the derivative of AID-1, AT11 [4] (Figure 1).

It is known that any extensions from the GQ structure, like single stranded short oligonucleotides, affect the GQ internal structure, stability, and properties [19], not to mention an extension with a massive module, such as another GQ. In addition, the existence of three lateral loops could interfere with the tight packing of two GQs of HD1s joined by a short link with a single T. Indeed, a putative structure of bi-HD1 does not correspond to a sum of two initial modules; probably the structure of the second module extending from the 3′-end of the first one is slightly different from HD1 due to CD spectroscopy (Figure 2A) [14]. Therefore, the structure of bi-modular GQ constructs should be carefully studied before functional assays.

The CD spectrum of HD1 and bi-HD1 (Figure 2) correspond to a topology of anti-parallel GQ [20]. The molar CD at 294 nm for bi-HD1 with K^+^ was not two times, but just 1.3 times more than that for HD1, which reflects the existence of two unequal GQs [14]. The CD spectrum of AID-1-T and bi-(AID-1-T) correspond to the topology of parallel GQ [20]. The molar ellipticity at 263 nm for bi-(AID-1-T) with K^+^ was exactly two times more than that for AID-1-T, which reflects the existence of two equal GQs, unlike for bi-HD1. These structural differences of the two twins could be due to the different orientation of the two GQs within the molecule. In bi-HD1 the two GQs could not stack because of the lateral loops; on the contrary, in bi-(AID-1-T) the two GQs could perfectly stack on each other [39]. Three-quartet AID-1-T and bi-(AID-1-T) could fold more effectively than two-quartet HD1 and bi-HD1 (Figure 2). This could be due to either the number of quartets within GQ, or the stacking within the twin mentioned above, or both.

As far as the effects of cation nature on the folding are concerned, generally, bi-modular constructs were less sensitive to the nature of the cation (Figure 3). In Na^+^, HD1 was, as expected, not well-folded (Figure 3A) [29], while bi-HD1 was almost folded, as it folds in favorable K^+^ (Figure 3B). For AID-1-T and bi-(AID-1-T) this effect was much more pronounced (Figure 3D,E); the introduction of Ba^2+^ stressed this effect. The folding of covalently joined GQs of bi-HD1 was affected by Ba^2+^ much more strongly than a single module of HD1 alone (Figure 3A,B), which supports the idea of unequal, imperfect GQs in bi-HD1. This was not that notable for AID-1-T and bi-(AID-1-T) because of the perfect GQ folding, and probably due to the reasons discussed above.

The thermal stability of a folded GQ strongly affected its functional activity, because most of tests were performed at 37 °C (310° K). After 35 °C (308° K) the thermal unfolding of HD1 was similar to that of bi-HD1 (Figure 5A), indicating some autonomy of GQ behavior beyond this temperature. However, an aT*m* about 40 °C (313° K) could not provide totally folded molecules. Parallel GQs of AID-1-T and bi-(AID-1-T) were much more thermally stable than anti-parallel GQs, having aT*m* above 75 °C (348° K), and providing almost totally folded molecules, which was firmly in favour of its applicability in exploring the anti-proliferative activity of these GQs. The difference in the thermal stability of mono- and bi-modular GQs was more pronounced. Mono-modular AID-1-T gave a non-covalent dimer via the stacking interaction of two molecules. It showed bi-phase thermal unfolding, a first gradual slow decrease of ellipticity, probably due to dissociation of dimers, and then the unfolding of the GQs themselves.

For testing the anti-proliferative activity, a rather high concentration of GQs is usually applied to cell cultures, i.e., micromole range. Therefore, an examination of the conformational/oligomeric homogeneity of GQs at these concentrations has to be carefully performed.

AS1411 behaves as a dimer, showing a slightly asymmetric peak (Figure 6C). For AS1411 the existence of a dimer fraction has been found [11], which could be due to the suggested bi-modular structure (Figure 1).

It is interesting to compare the migration of bi-HD1 with that of AS1411. The first twin, being less compact because of the lateral loops, goes faster than the putative second one, which again indirectly supports the suggested bi-modular structure of AS1411.

It is no surprize that the migration of bi-HD1 is much faster than mono-modular HD1. Bi-modular bi-(AID-1-T) exhibited approximately the same mobility as the non-covalent dimer of mono-modular AID-1-T; the dimerization ability of the later has also been observed before [16]. The introduction of Ba^2+^ stimulated further dimerization of the non-covalent dimer into a putative tetramer.

As was already mentioned, GQs have attracted a great deal of attention as potential anti-proliferative agents [1]. To increase the GQs activity, a variety of modifications have been applied, mainly attempting to improve their chemical stability in the milieu.

Aside from that trend, in this research we continued to explore an approach of constructing twin molecules by the covalent linking of identical functionally active GQ modules. Even for HD1 with lateral loops, it was shown that making a bi-modular twin molecule was effective for creating an anti-proliferative agent for the neural cancer cell line U87 (Figure 7A,B) [14].

Parallel GQs with propeller loops have the folding properties discussed above, and due to this they are more suitable for exploring anti-proliferative activity. GQ of AID-1-T is known for its ability to bind several GQ-binding proteins, like HIV integrase, and IL-6 receptor [25], but its anti-proliferative activity has not been explored toward cancer cells, nor neural cancer cells in particular.

At the beginning, all five GQs were tested toward the neural cancer cell line, U87, applying three different concentrations of GQ: 0.1, 1.0, and 10 μM, in an attempt to find the most active GQ, as well as to compare mono-modular and bi-modular constructs (Figure 7). As a control for a non-cancer cell line, human embryo fibroblasts were used. First, all GQs, except HD1, at 10 μM had a reduced U87 cell viability, and only bi-modular GQs were active at 1 μM, which stressed the value of making twin molecules. There were no effects on the human embryo fibroblast cell line at any concentration tested.

Unique results were observed when the primary glioma cell cultures from patients were tested. Brief data on patients are listed in Table 1. Six cell cultures were tested, and it turned out that the viability of just three cell cultures, G01, Sus/fP2, and G11, were affected to a very different extent (Figure 8). The IC50 for the G01 cell culture was >10 μM; the Sus/fP2 and G11 cell cultures were 1–10 μM; and only in one case, for bi-(AID-1-T) toward G11 cell culture, the IC50 was less than 1 μM. In general, the most sensitive cell culture was G11, and the most active GQ was bi-(AID-1-T) (Figure 8B).

Therefore, the conclusion was the following: the anti-proliferative activity of different GQs toward different cell cultures was different. This ambiguity could be solved in two ways: from the GQ structure, and from the cell nature.

**Figure A2:**
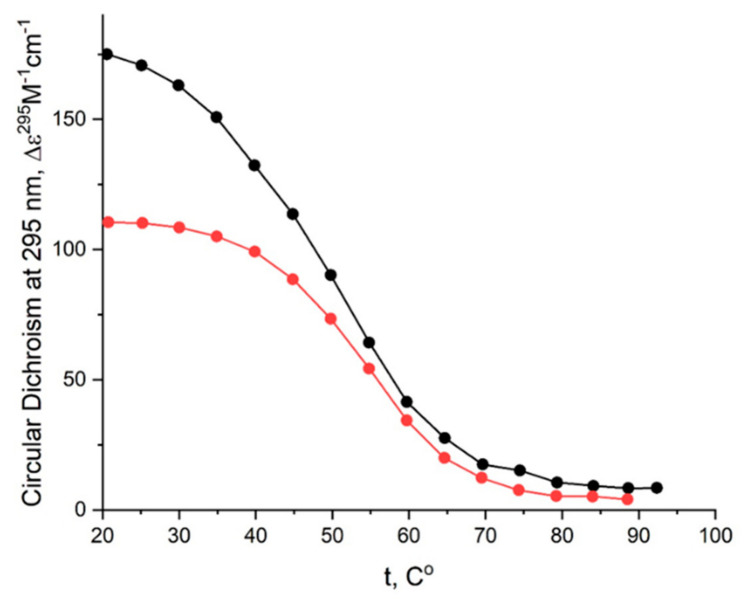
CD melting profile of GQs under study in the buffer C (140 mM NaCl, 10 mM KCl, 5 mM BaCl_2_): HD1 (red), bi-HD1 (black).

**Figure A3:**
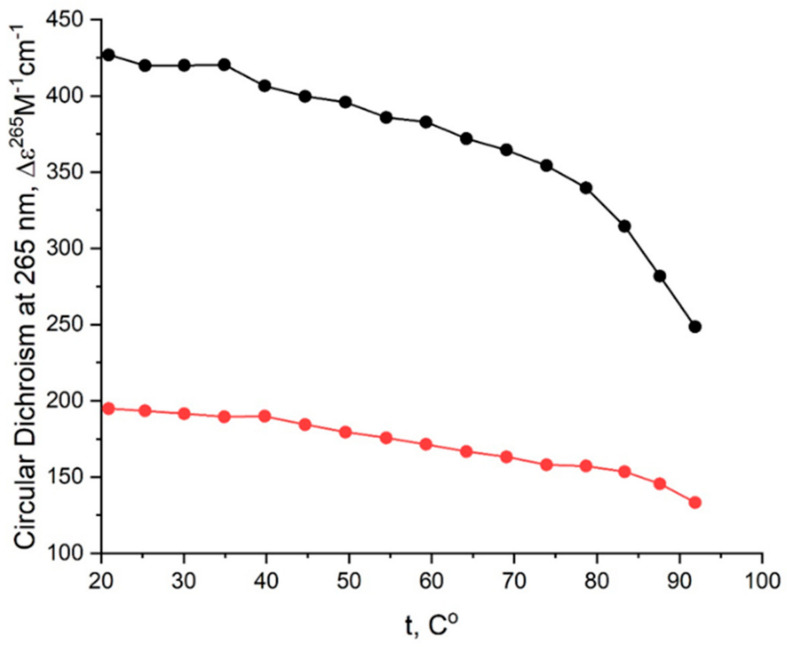
CD melting profile of GQs under study in the buffer C (140 mM NaCl, 10 mM KCl, 5 mM BaCl_2_): AID-1-T (red), bi-(AID-1-T) (black).

**Figure A4:**
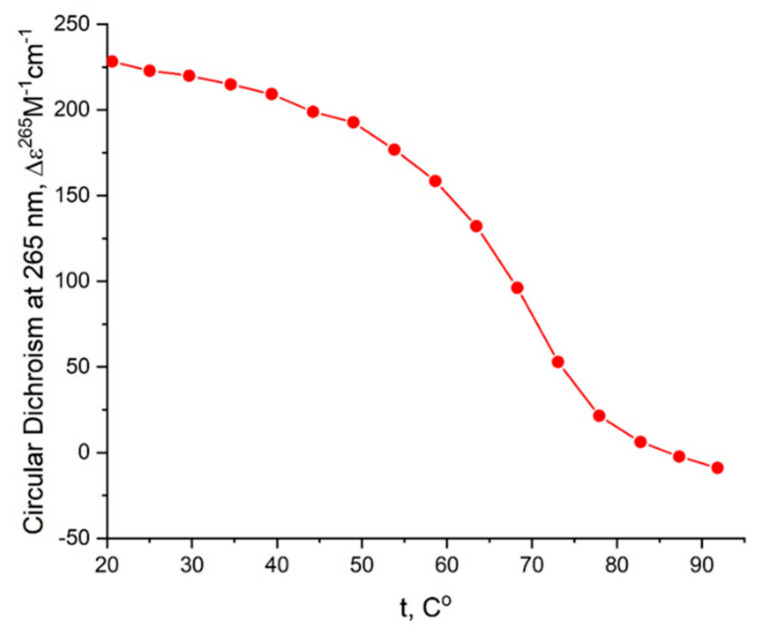
CD melting profile of GQs under study in buffer C (140 mM NaCl, 10 mM KCl, 5 mM BaCl_2_): AS1411 (red).

The specific GQ structure governs the type of action on a cancer cell line; changes in GQ conformation affect its activity: it could either reduce/abolish activity [14] or enhance activity (Figure A2, Figure A3, Figure A4 ). The inversion of chain polarity and usage of unnatural nucleotides could increase anti-cancer activity [15,40,41], but the exact reason, like changing the conformation only, has to be established properly in each case.

The phenomenon of inter-tumor heterogeneity, in terms of the response to specific agents, like antibodies, is not surprising [36], and needs to be studied in more detail to attempt to find reasons. GQs, as crypto-aptamers, could be considered as relatively specific agents as well, though less specific than antibodies, because they can attack not a single protein, but a group of proteins, like GQ-binding proteins. This suggestion was correct in the case of AID-1-T GQ, which could interact with different proteins: the receptor IL-6R, and HIV integrase [25]. Therefore, more studies are required to find clues to the mechanism of action of its twin, bi-(AID-1-T).

At this stage of the research, some general questions could be addressed, whether this intertumor heterogeneity toward GQs is due to the very first steps of up-take, endocytosis activity, due to the status of the transport protein, like nucleolin, or due to the status of unknown receptor; or whether it reflects different inner-cell events: the pattern/status of GQ-binding proteins. Any correlations between possible histology/pathology and the molecular biology features of real patients and GQs activities remain to be established.

## 4. Materials and Methods

Conventional human cell lines U87 and fibroblasts from human embryo were provided from the collection of the Centre of Neurosurgery (Moscow, Russia). GBM cultures N1, G11, Sus/fP2, G22, G23, and G01 were developed in the Centre of Neurosurgery from the surgery samples of patients (Burdenko National Medical Research Centre of Neurosurgery). The data on GBM grade and some patient details are summarized in Table 1; all samples had WT IDH1 (isocitrate dehydrogenase 1). This study was approved by the Ethics Committee of Burdenko Neurosurgical Institute, Russian Academy of Medical Sciences (№_12/2020). All subjects gave written informed consent in accordance with the Declaration of Helsinki.

### 4.1. Materials and Reagents

NaCl and BaCl_2_ Merck (KGaA, Darmstadt, Germany); KCl Serva (Heidelberg, Germany), HCl Bio–Rad Laboratories (Hercules, CA, United States), Tris Invitrogen (Carlsbad, CA, USA), NaH_2_PO_4_, Na_2_HPO_4_∙2H_2_O, glycerol PanReac AppliChem (Barcelona, Spain), acetonitrile HPLC grade Sigma–Aldrich (St. Louis, MO, USA), dimethylsulfoxide PanEco (Moscow, Russia), growth medium DMEM/F–12, PBS Gibco, Thermo Fisher Scientific (Carlsbad, CA, USA), MTT–reagent CellTiter 96^®^ Aqueous Promega (Madison, WI, USA). Solutions were made with deionized water, Milli–Q (Merck MilliPore, Burlington, MA, USA), and were filtered prior to experiments though 0.2 µm nitrocellulose filters, Sarstedt (Nümbrecht, Germany).

Buffer composition: buffer A: 20 mM Tris-HCl, pH 7.1, 140 mM NaCl; buffer B: 20 mM Tris-HCl, pH 7.1, 140 mM NaCl, 10 mM KCl; buffer C: 20 mM Tris-HCl, pH 7.1, 140 mM NaCl, 10 mM KCl, 5 mM BaCl_2_; buffer D: 20 mM Tris-HCl, pH 7.1, 140 mM NaCl, 10 mM KCl, 1% (*v*/*v*) glycerol.

All oligonucleotides were synthesized and HPLC-purified by Evrogen Ltd. (Moscow, Russia).

The sequences of GQs are listed in Table 2. The oligonucleotides were named according to their first appearance in the literature. Four of the six oligonucleotides had been described previously. Bi-modular GQ, bi-(AID-1-T) was designed for the first time.

### 4.2. Preparation of GQ Solutions

Molar extinction coefficients of oligonucleotides were calculated using the nearest-neighbor model [42]. Optical density was measured using a UVI photometer BioPhotometer 6131 from Eppendorf (Hamburg, Germany). GQs were dissolved in buffers. All solutions were heated at 95 °C for 5 min, and cooled at room temperature.

### 4.3. Circular Dichroism Spectroscopy (CD)

CD spectra (as well as UV spectra) were recorded using a CD spectrometer Chirascan from Applied Photophysics (Leatherhead, UK) and a Dichrograph MARK-5 (Jobin-Yvon; Montpellier, France) equipped with a thermoelectric temperature regulator. Quartz cuvettes had a 1-cm optical path length. CD and UV spectra were measured in the wavelength range 220–340 nm, and temperatures range 20–95 °C, with a 5 °C step; the samples had a thermostatic pause for 7 min. Spectra of buffer solution were subtracted as baselines. Melting temperatures were calculated from CD melting profiles at 265 nm for parallel GQ, and at 295 nm for antiparallel GQ [43].

### 4.4. Size-Exclusion HPLC

Size-exclusion chromatography (SEC) was conducted as described [34,35] using an Agilent 1200 HPLC system with an autosampler and diode array detector (Agilent; Santa Clara, CA, USA). The HPLC column was a TSKgel G2000SWXL (Tosoh Bioscience; King of Prussia, PA, USA), 30 cm length, 0.78 cm diameter, 5 µm particle diameter, 12.5 nm pore diameter; the column was conventionally recommended by the manufacturer for the separation of proteins with MW (molecular weight) in the range 5–150 kDa. The developed conditions for the separation of oligonucleotides were the following: temperature 25 °C, mobile phase water/acetonitrile 9:1 *v*/*v* with potassium phosphate buffer (60 mM KH_2_PO_4_ and 140 mM K_2_HPO_4_, pH 6.9), and flow rate 0.5 mL/min. Absorption at 260 nm was registered with 10 nm bandwidth.

The column was calibrated with a set of duplexes (GeneRuler Ultra Low Range DNA Ladder from Thermo Fisher Scientific (Carlsbad, CA, USA). The linear range for log (MW) from Vr/V0 was achieved for duplexes with 10–100 base pairs (Vr—retention volume, V0—dead volume of the column, 5.64 mL). The following equation was acquired from the calibration experiment and used for further calculations of apparent molecular weights of GQ and complexes: log(MW) = 6.51 − 1.67 · Vr/V0.

### 4.5. MTT Assay

The MTT assay estimated cell viability, which was measured after adding GQ; the standard protocol was used [44]. Cells were placed in 96-well plates; 1500–2000 cells per well were supplied with 200 µL of growth medium, and cultivated for 48 h under standard conditions (37 °C, 5% CO_2_, and controlled humidity). Then the growth medium was removed and 100 µL of fresh growth medium was added with GQ in the appropriate content; five repeats for each sample were made. Cells were incubated for 72 h; then growth medium was removed; the cells were washed with PBS; 100 µL fresh growth medium and 20 µL of MTT reagent were added for 2 h. Growth medium without cells and MTT-reagent was used as a baseline sample. Optical density was measured using Infinite M200 Pro (Tecan, Switzerland) at 490 nm.

## 5. Conclusions

G-quadruplex DNA oligonucleotides (GQs) exhibited specific anti-proliferative activity for human cancer cell lines. This ability could be translated into the treatment of glioblastoma multiform (GBM), which has a poor prognosis and low-efficiency therapeutics. A novel bimodular GQ, bi-(AID-1-T), a covalent twin of the previously described three-quartet AID-1-T, was structurally and functionally characterized. A comparison of bi-(AID-1-T) with its module, AID-1-T, and bi-modular bi-HD1 with its module two-quartet HD1, and conventional AS1411, was made. Bi-(AID-1-T) had the highest anti-proliferative activity for neural cancer cell line U87, while not affecting the control human embryonic fibroblasts. For the first time GQs were tested toward several primary GBM cultures from patient surgical samples. The sensitivity of patient primary GBM cultures toward GQs varied, with an apparent IC50 less than 1 μM for bi-(AID-1-T) toward the most sensitives G11 cell culture (GBM, Grade III). 

## Figures and Tables

**Table 1:** List of patient glioma primary cell cultures, and brief data on patients. All samples have wt IDH1 (wild type of isocitrate dehydrogenase 1, a conventional glioma marker).

Cell Culture	Glioma Grade	Patient Gender	Patient Age
G11	III	F	33
Sus\fP2	IV	F	60
G01	IV	M	39
G23	IV	M	53
G22	IV	F	36
N1	IV	M	54

**Table 2:** List of GQ sequences of DNA oligonucleotide under study. Proposed sequences of linkers of GQ modules are underlined. References for known GQ are in the text.

GQ	DNA Sequence
AS1411	GGTGGTGGTGGTTGTGGTGGTGGTGG
HD1	GGTTGGTGTGGTTGG
bi-HD1	GGTTGGTGTGGTTGGTGGTTGGTGTGGTTGG
AID-1-T	GGGTGGGTGGGTGGG
Bi-(AID-1-T)	GGGTGGGTGGGTGGGTTTGGGTGGGTGGGTGGG

**Figure A5:**
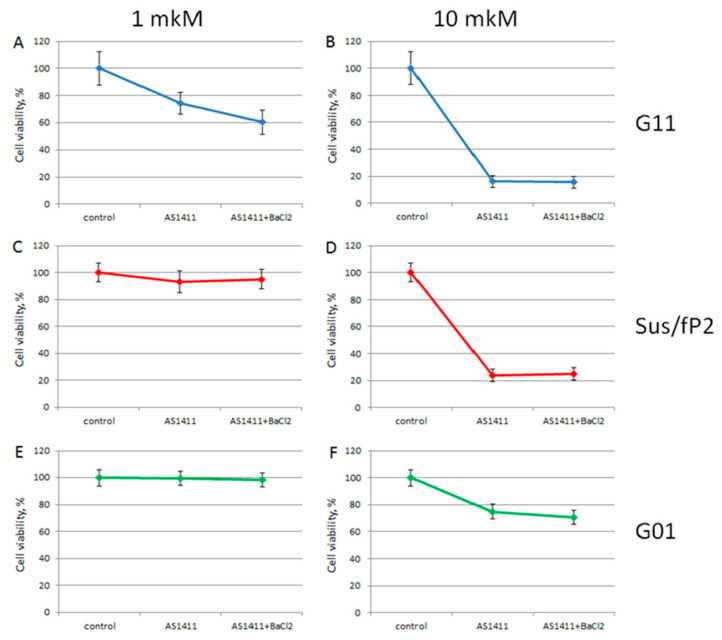
MTT assay data on cell viability of three patient primary glioma cell cultures after treatment with AS1411+Ba^2+^ at 1 and 10 μM, for 72 hrs. Patient primary glioma cell cultures: G11 (**A**,**B**, blue), Sus/fP2 (**C**,**D**, red), G01 (**E**,**F**, green). Data are shown as (mean +/− SD). Patient data are in Table 1.

**Figure A6:**
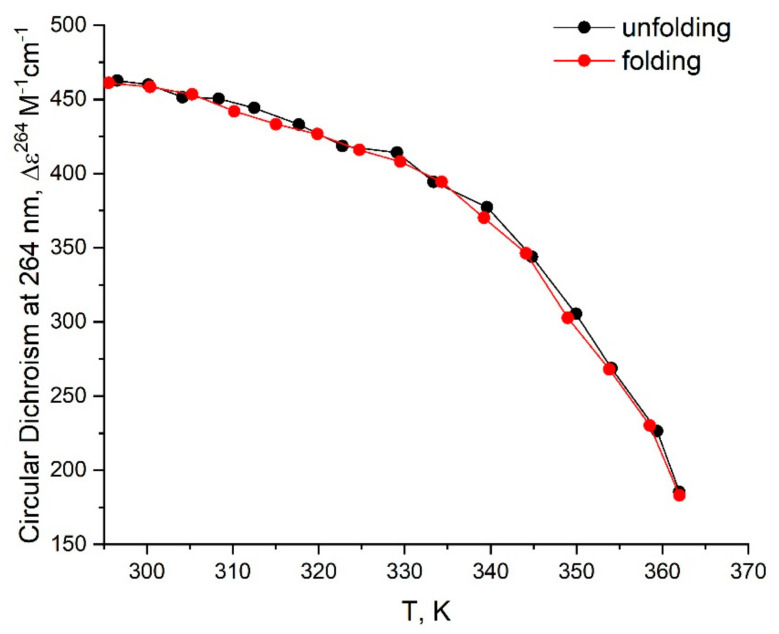
CD unfolding (black) and folding (red) profiles for bi-(AID-1-T) in buffer B (140 mM NaCl, 10 mM KCl; (Na^+^, K^+^).

## Data Availability

The data presented in this study are available on request from the corresponding author. The data are not publicly available due to the reason that some of the generated data is not published.

## Appendix A

Previously it has been shown by us, that the coordination of Ba^2+^ cation by GQ of bi-HD1 stabilized GQ and abolished its anti-proliferative activity [14].

Owing to the conformational heterogeneity of AS1411, it was suggested to stabilize a conformation of AS1411 with Ba^2+^ coordination in buffer C (Figure 3, Figure A1 and Figure A4), and study the anti-proliferative activity of this complex toward four primary glioma cell cultures (Figure A5). Contrary to bi-HD1, the activity of AS1411+ Ba^2+^ was increased. The most demonstrative example was the treatment of the most sensitive G11 culture with 1 µM AS1411+ Ba^2+^, which was more effective than AS1411+K^+^ (Figure A5).
